# Keys to female athlete performance in Paris 2024

**DOI:** 10.3389/fpsyg.2025.1718337

**Published:** 2026-01-09

**Authors:** Emre Boz, Eda Adatepe, Hüsniye Çelik, Hakan Kırkbir, Tuncay Öktem, Yılmaz Ünlü, Ümit Öz, Büşra Yılmaz

**Affiliations:** 1 Department of Physical Education and Sports, Faculty of Sport Sciences, Bayburt University, Bayburt, Türkiye; 2 Department of Sports Management, Faculty of Sport Sciences, Bayburt University, Bayburt, Türkiye; 3 Karadeniz Technical University, Trabzon, Türkiye; 4 Bolu Abant Izzet Baysal University, Bolu, Türkiye

**Keywords:** athlete, female, Olympic Games, Paris 2024, sports performance

## Abstract

The research was conducted to identify the key factors influencing the performance of Turkish female athletes participating in the Paris 2024 Olympic Games. In this context, seven female Olympic athletes who participated in the relevant organization volunteered to take part in the research. A semi-structured interview form prepared by the researchers was used to identify the participants’ views on the factors affecting their performance. Individual interviews were conducted with each participant at different times, and all interviews were recorded. All interview transcripts obtained were analyzed and visualized using MAXQDA 2020 software. In the study, the experiences of female athletes were grouped under five themes: factors most affecting performance, environmental factors, physiological factors, socio-cultural factors, and psychological factors. The findings show that the athletes’ experiences are not limited to athletic performance alone, but are also influenced by multidimensional factors such as organizational shortcomings, environmental conditions, psychological pressure, socio-cultural interactions, and physiological conditions. In the study, first-time participants were positively influenced by motivation, team support, and communication with foreign athletes, while experienced athletes, having higher expectations, criticized factors such as organizational shortcomings, hygiene, and nutrition issues. The study reveals that the factors affecting the performance of female athletes are multidimensional and interactive, emphasizing the importance of comprehensively considering physiological, psychological, environmental, and socio-cultural needs in the planning of international sports organizations. In particular, strengthening health support, nutrition services, cultural adaptation, and social support mechanisms are recommended as measures that will increase athletes’ performance and experience satisfaction.

## Introduction

The largest and most prestigious sporting event on a global scale is the Olympic Games, and for this reason, these events feature content that captures the attention of the entire world (Chalkley and Essex, 2010). This sports organization, whose roots date back to ancient times, has been influenced by various cultural and political factors throughout history (Tekin and Tekin, 2014). For example, the 1936 Berlin Olympics were used by Nazi Germany for propaganda purposes, which contradicted the universality of sport (Gülada, 2019). The 1980 Moscow Olympics were boycotted by the United States and some allied countries due to the Soviet Union’s invasion of Afghanistan, demonstrating that sports were overshadowed by political tensions (Jefferys, 2012). From a cultural perspective, the 1964 Tokyo Olympics served as a means for Japan to showcase its post-war renaissance and modernization to the world (Öncü, 2023). Through these examples, it is thought that the Olympic Games play an important role in many factors beyond being a platform for showcasing sporting achievements.

Events that have shaped the agenda and left lasting impacts in the history of the Olympic Games continue to occur today, just as they did in the past. The most prominent example of this is the Olympic Games organized in Paris, the capital of France, in 2024 (Ayverdi et al., 2025). This is because the Paris 2024 Olympics have become the focus of cultural, religious, and political debates, going beyond being merely a sporting event (Voinea et al., 2024; Brocherie et al., 2024; Liu, 2024). Among these incidents, which were considered scandalous, many topics attracted the attention of the entire world, ranging from controversial moments at the opening ceremony to debates on gender identity, the hijab issue, and environmental pollution. Among all these developments, the most notable elements are the practices and debates concerning female (Demircioğlu-Biricik, 2024).

The difficulties and obstacles that female face in participating in the Olympic Games in various identities such as spectators, athletes, coaches, managers and board members date back to the ancient Olympics (Emery, 1984), and this situation has continued for many years and continues to exist today. These events, which are inconsistent with the principles of Olympism, affect athletes in many ways and also impact their athletic performance (Köse, 2025). Research in this area indicates that political, socio-cultural, and environmental factors can directly and indirectly influence athletic performance (Romi et al., 2023; Begović et al., 2021), the contributions of social, physical, and psychological factors to athlete motivation and performance (Zainuddin et al., 2023), stress sources at mega sporting events and the effects of this stress on athletes (Gould et al., 2002; Pensgaard and Duda, 2003), the relationship between media pressure and stress, anxiety, and performance in elite athletes (Kristiansen et al., 2011), and the effects of cultural interaction on athletes in major sporting events (Girginov and Hills, 2023).

In addition to the physical and mental factors that influence athletic performance, it is also intriguing to explore which factors play a role in major events such as the Olympic Games and what influences the athletic performance of female athletes in a high-profile event like the Paris 2024 Olympic Games. In this context, the main objective of the research is to examine the key factors affecting the performance of female athletes at the Paris 2024 Olympic Games. In light of the research objective, answers have been sought primarily to the following questions:

1. What are the key factors most affecting the performance of female athletes at the Paris 2024 Olympic Games?
2. What are the physiological factors affecting the performance of female athletes at the Paris 2024 Olympic Games?
3. What are the psychological factors affecting the performance of female athletes at the Paris 2024 Olympic Games?
4. What are the environmental factors affecting the performance of female athletes at the Paris 2024 Olympic Games?
5. What are the socio-cultural factors affecting the performance of female athletes at the Paris 2024 Olympic Games?

## Research method

In this study, the qualitative “case study” method, which enables social phenomena to be deeply understood and investigated within their respective contexts, was used in accordance with the research objective. As mentioned above, traditional methods such as survey studies offer a limited and predefined perspective. The purpose of this research is to understand the factors affecting the performance of female athletes participating in the Paris 2024 Olympic Games and how they experience these factors during the games, as reflected in the athletes’ own voices.

This research, designed as a case study, takes into account the criteria emphasized by Merriam (2009). Accordingly, case studies aim to reflect the reality of a specific phenomenon, contribute to the understanding of different factors by focusing on the questions of “how” and “why,” and increase the applicability of the results obtained. Similarly, Saldana (2023) also states that the fundamental goal of case studies is to produce systematic and in-depth knowledge on the research topic. The reason for selecting the Paris 2024 Olympic Games as a single case is that it reflects the reality of a specific phenomenon. In other words, it is a global sports organization with a long-standing historical, institutional, and cultural background where multifaceted experiences are lived. The findings were evaluated within this framework, and key factors were identified and discussed. On the other hand, female athletes having similar athletic backgrounds influenced their participation experiences.

## Participants and sampling

The qualitative method used in the research aims to provide an in-depth and multifaceted perspective on the subject. Participants were selected using the criterion sampling technique, one of the purposive sampling methods. With criterion sampling, the researcher(s) can determine criteria and select participants according to the focus of the study. Thus, individuals who meet the criteria set for the sample are included in the research group (Büyüköztürk, 2009). Therefore, Turkish female athletes participating in the Paris 2024 Olympic Games were selected as the research group.

With the approval of the research ethics committee, female athletes participating in the Paris 2024 Olympic Games were informed about the purpose and process of the research. During this process, female athletes who might be willing to participate in the research were contacted. As a result, the study group consisted of seven (7) female athletes (mean age = 27.85; Sd = 3.436) representing Turkey at the Paris 2024 Olympic Games. It is believed that the number of interviews is sufficient for this qualitative case study (Creswell, 2013; Marshall et al., 2013). As this research employs the qualitative case study method, the determination of the sample size (*n* = 7) was not based on the statistical power required for generalization, as is the case in quantitative studies; instead, the guiding principle was the achievement of analytical saturation (thematic saturation). The size of our purposeful sample (*n* = 7) was deemed sufficient because, after the sixth and seventh interviews, no new significant themes, categories, or novel insights emerged regarding the factors affecting the performance and experiences of female athletes at the Paris 2024 Olympic Games, thus providing a rich, detailed, and holistic understanding of the single case, which is the primary objective of a qualitative case study (Merriam, 2009; Saldana, 2023). We considered data saturation to be achieved when the researchers determined that all dimensions of the research question were fully explored, and the data began to show redundancy and replication, leading to theoretical completeness within the context of the study’s focus (Saldana, 2023); the seven in-depth interviews allowed us to build robust themes and cross-case comparisons within the context of the Paris 2024 Games. Therefore, we assert that the final sample size of *n* = 7 was appropriate and sufficient to reach analytical saturation, providing a comprehensive and in-depth understanding of the central phenomenon, aligning with the standards for rigorous qualitative case study research. The athletes in the sample compete in boxing, wrestling, athletics, taekwondo, and fencing. Furthermore, concealing the identities of participants in qualitative research is one of the most fundamental requirements of research ethics. This practice is important in terms of protecting the privacy of participants, minimizing social or institutional risks, and ensuring that individuals can express their opinions safely (Creswell, 2013). Ethics committees and international standards (e.g., the World Medical Association, 2013) require researchers to ensure that personal information is not disclosed and that data is used solely for research purposes. Therefore, participants are often identified by codes or pseudonyms, ensuring that experience and meaning take precedence over identity in the research (Patton, 2015). In this study, female athletes were referred to as FA1, FA2, FA3, and so on.

The Paris 2024 Olympic Games were held from July 26 to August 11, 2024, with 204 countries participating. Competitions were held in 37 sports disciplines across 35 different venues. Turkey was represented by 101 athletes in the event, which featured a total of 10,500 athletes. Of these athletes, 54 were female and 47 were male. Across the Olympics as a whole, approximately 5,250 female and 5,250 male competed (Turkish National Olympic Committee, 2025).

## Data collection and analysis

In the study, a semi-structured interview prepared by researchers was used to identify the factors that influence the performance of female athletes. Researchers reviewed the relevant literature and developed an interview form consisting of five questions. Accordingly, the interview questions covered the factors that most affected the performance of female athletes participating in the Paris 2024 Olympic Games, including physiological, psychological, environmental, and cultural factors. Qualitative research principles were used in preparing the questions. Before starting the study, the researchers obtained the evaluation of two experts in the field to ensure the comprehensibility and validity of the interview questions. After the ethics committee approval was received 11 days after the end of the Paris 2024 Olympic Games, a pilot study was conducted with a single participant and the measurement tool was finalized based on the results obtained. With the participants’ consent, the interviews were audio recorded and transcribed for analysis. Thematic analysis was applied in the study to examine the experiences of female athletes participating in the Paris 2024 Olympics. First, the transcribed audio recordings of the interviews were carefully read, and the data set was generally recognized. Then, the participants’ statements were coded by separating them into units of meaning. During the coding process, recurring and meaningful patterns were identified, and these codes were grouped under themes based on their similar content. To ensure coding consistency and enhance the dependability of the findings, a validation strategy was employed. Researcher A was primarily responsible for coding the entire dataset, but Researcher B independently reviewed and validated all codes and theme assignments for a selected portion of the transcripts (e.g., 30% of the data). Discrepancies were resolved through mutual discussion and consensus building until 100% agreement was reached on the meaning and application of all codes. This peer debriefing and validation process strengthens the trustworthiness of the analysis and assures that the themes accurately reflect the participants’ voices (Guba and Lincoln, 1989; Merriam, 2009; Spall, 1998). The themes created were interpreted in relation to the research questions, enabling a systematic understanding of the factors affecting the performance of female athletes. This approach allowed for a deep understanding of the participants’ experiences and enabled reliable inferences to be made based on qualitative data. All views obtained were analyzed using MAXQDA 2020, a data analysis software developed for qualitative and mixed-method research.

## Findings

**Figure 1:**
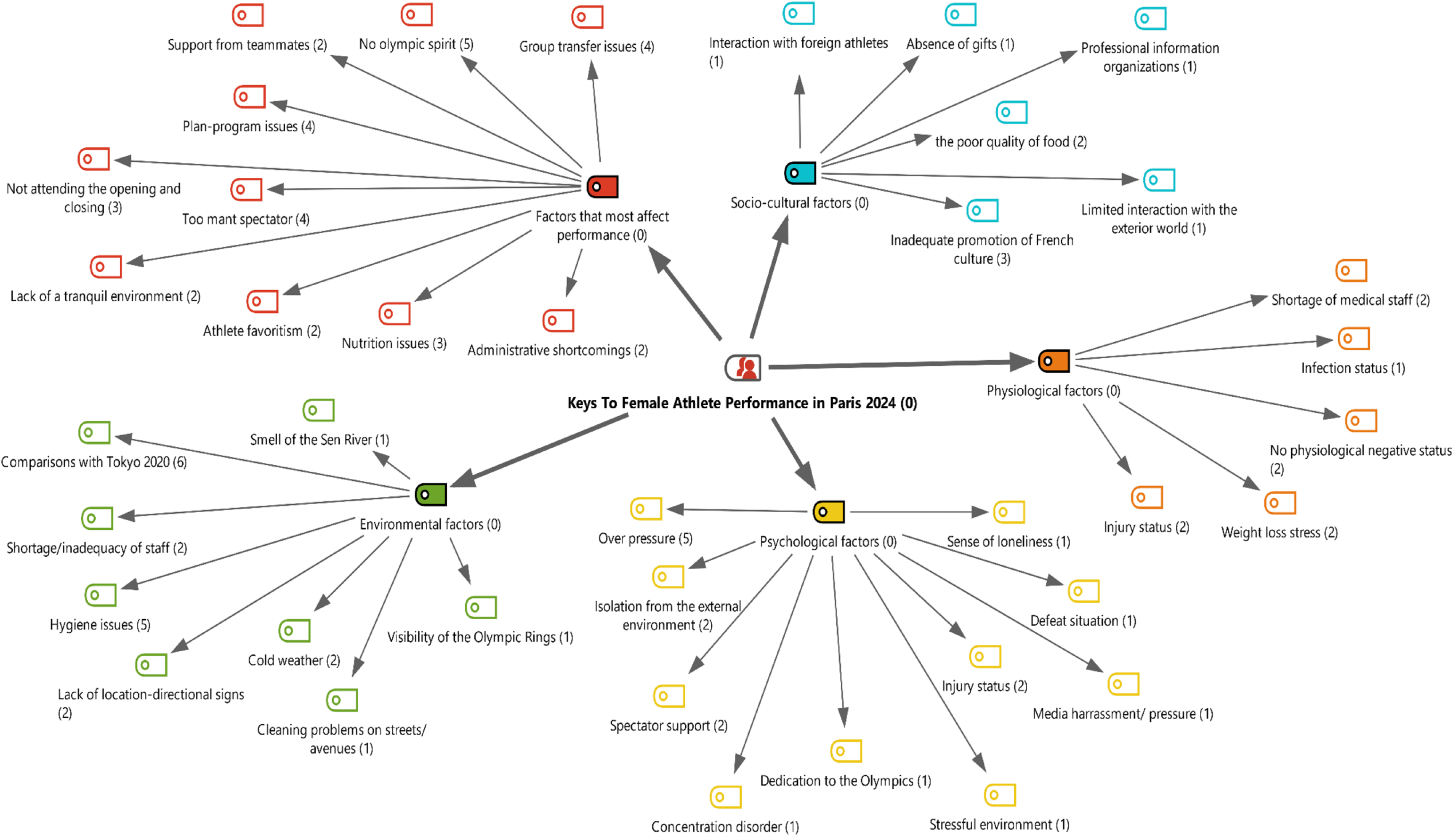
Hierarchical code-subcode model.

The research findings are presented descriptively, taking into account the theoretical framework and using direct quotes from female athletes. The aim here is to convey the rich data obtained from in-depth interviews within a structured framework and to give space to the experiences of female athletes, whose voices are rarely heard in research related to the Olympics. Below, the findings of the key factors identified based on the theoretical framework are presented under five themes: “Factors most affecting performance,” “Environmental factors,” “Physiological factors,” “Socio-cultural factors,” and “Psychological factors.” (Figure 1).

**Figure 2:**
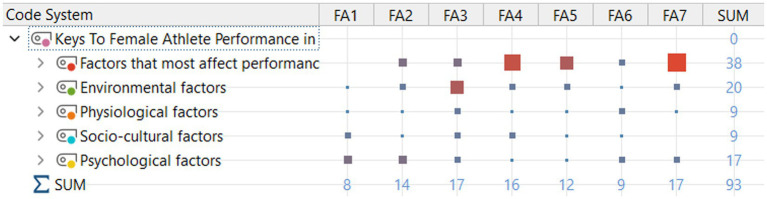
Table of participants’ opinion intensities by theme.

According to Figure 2, it has been observed that the factors affecting female athlete performance at Paris 2024 have a multidimensional structure. When examining coding frequencies, it is noteworthy that participants focused most on the theme of “factors most affecting performance” (*f* = 38). This finding shows that the elements determining athletes’ performance are frequently discussed in general framework. In addition, “environmental factors” (*f* = 20) and “psychological factors” (*f* = 17) were other areas that participants emphasized. In particular, it is understood that issues such as the training environment, climatic conditions, and psychological resilience are considered critical for performance. On the other hand, “physiological factors” (*f* = 9) and “socio-cultural factors” (*f* = 9) were emphasized less. This indicates that participants considered these two dimensions to be of secondary importance in terms of performance. When examined on a participant basis, the highest contribution was made by KS3 (*f* = 17) and KS7 (*f* = 17), while the lowest contribution was provided by KS1 (*f* = 8) and KS6 (*f* = 9). Overall, the findings reveal that perceptions of female athlete performance are particularly concentrated in the environmental and psychological dimensions.

**Figure 3:**
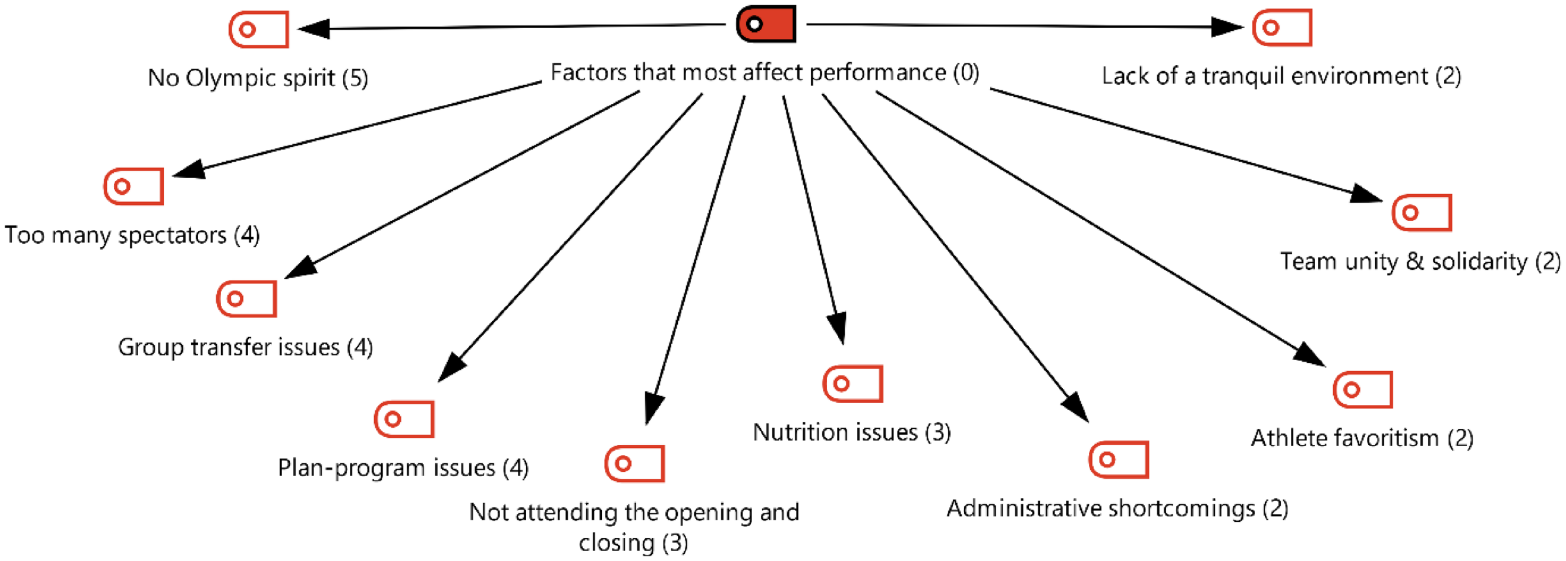
Theme 1—factors that most affect performance.

Figure 3 presents codes related to the factors that most affected performance under theme 1. Participants reported the presence of elements such as a lack of Olympic spirit, spectator density, fragmented movement of delegations, nutrition problems, and managerial deficiencies under this theme. Participants’ views are presented below:

*“I said that European countries would organize a beautiful event in a city like Paris, both artistically and socially, but I don't know if it was because I had such high expectations, but I was very disappointed. I never felt like we were in an Olympic mood. (KS7).”*

*“…first of all, I think the biggest problem was the food. There wasn't much variety, and there were always long lines when we wanted to get food. We had to wait in line. For example, I wanted to get something from the grill section, but there was such a long line that I had to rush to make it to practice. I didn't wait; I just went and got some snacks instead. (KS5).”*

*“We competed in Tokyo without spectators and in Paris with, and I can say that this had a significant impact on both the other athletes and me. (KS4).”*

**Figure 4:**
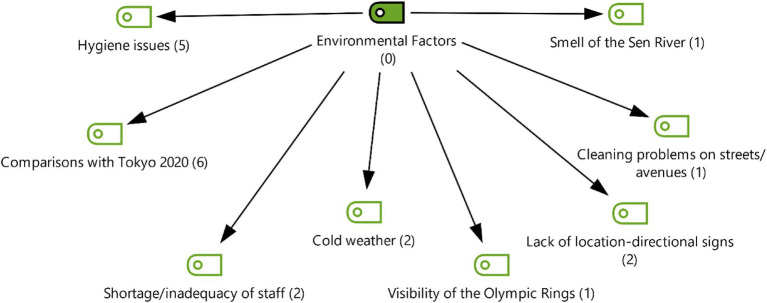
Theme 2—environmental factors.

Figure 4 shows Theme 2, which consists of environmental factors. Participants’ comments regarding comparisons with Tokyo, hygiene issues, lack of directional signage, and lack/inadequacy of staff are noteworthy. Some comments belonging to this category are listed below:

*“There really wasn't a clean environment; the environment didn't seem very clean or hygienic to me. The places where we ate had a strong odor. There was a mess in the place where we got our food, and I didn't like that (KS3).”*

*“They weren't very good with the staff either. We didn't have this problem in Tokyo because the staff could help you immediately, but I never saw this in Paris. (KS2).”*

*“No information was provided about transportation from the airport to the village. There were buses outside, and they said they would call us, so we waited there. As an athlete going to the Olympics, I would have liked a bigger welcome and safer transportation. We arrived at the village, and after arriving at the village, no one told us which building we were staying in or where the Turkish delegation was staying. We kept going to different places, trying to figure out how to get there. We tried to figure it out ourselves how we could get there. (KS3).”*

**Figure 5:**
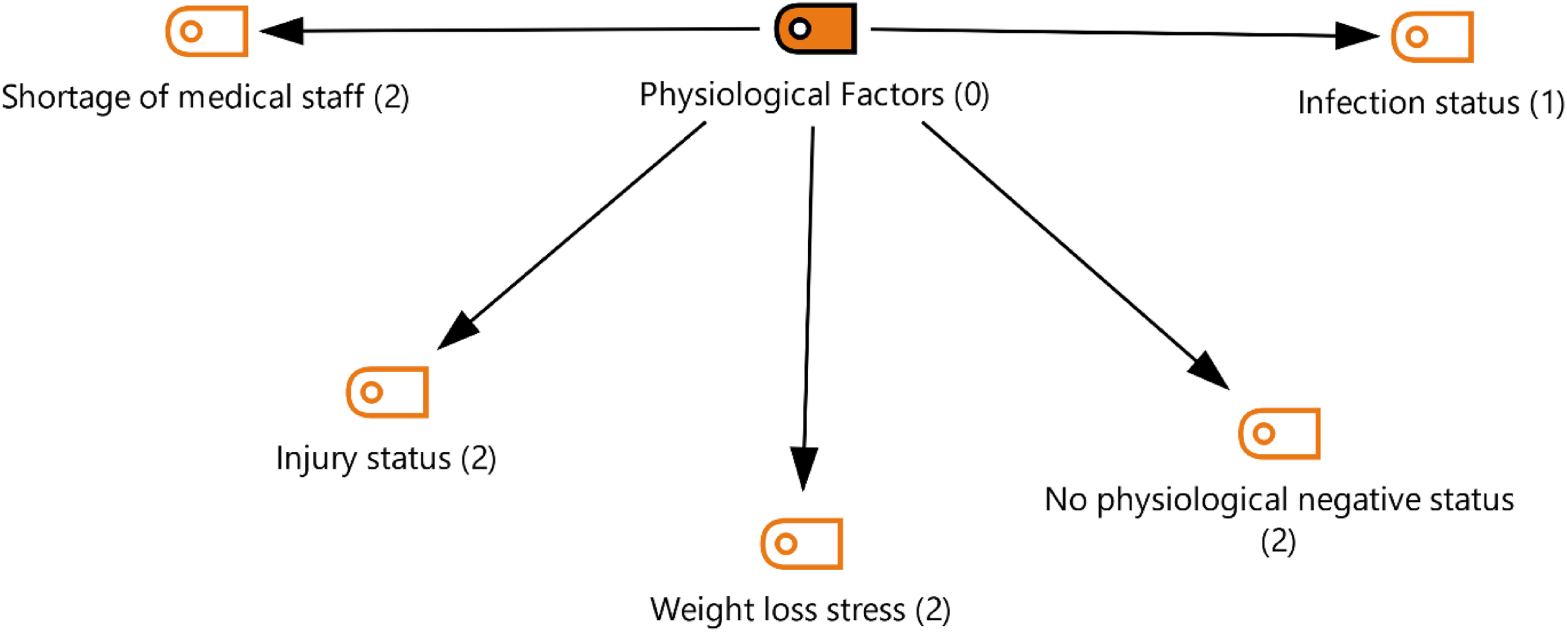
Theme 3—physiological factors.

Theme 3: Physiological factors are presented in Figure 5. When examining the theme of physiological factors, it is seen that female athletes’ views are included on topics such as insufficient medical staff, injury situations, weight loss stress, infection status, and the absence of physiological disadvantages:

*“The Turkish delegation had brought a medical team with them. The Turkish Ministry of Youth and Sports had set up an area there. There was a doctor and a physical therapist, but because there were so many athletes, they operated on an appointment system. Since I hadn't worked with a physical therapist or doctor before as an athlete, and because I'm a bit shy, I didn't feel comfortable. I always felt anxious during my treatment… (FA3).”*

*“During this period, athletes must be able to stay focused, concentrate, and maintain their weight. You have to stay at that weight for about 15-20 days, no matter how you look at it. And as stress increases towards the end, losing weight also becomes more difficult (FA2).”*

*“I suffered an injury close to the Olympics. I didn't have much time to recover. I was competing for a spot. I had to win that spot. I think I was physically affected because my body had to be ready, I had to win the spot, and I had my injury (FA6).”*

*“… I participated as a physiologically prepared athlete. I didn't allow anything negative to happen there. Frankly, it can't be said that there wasn’t anything that affected me greatly (FA1).”*

**Figure 6:**
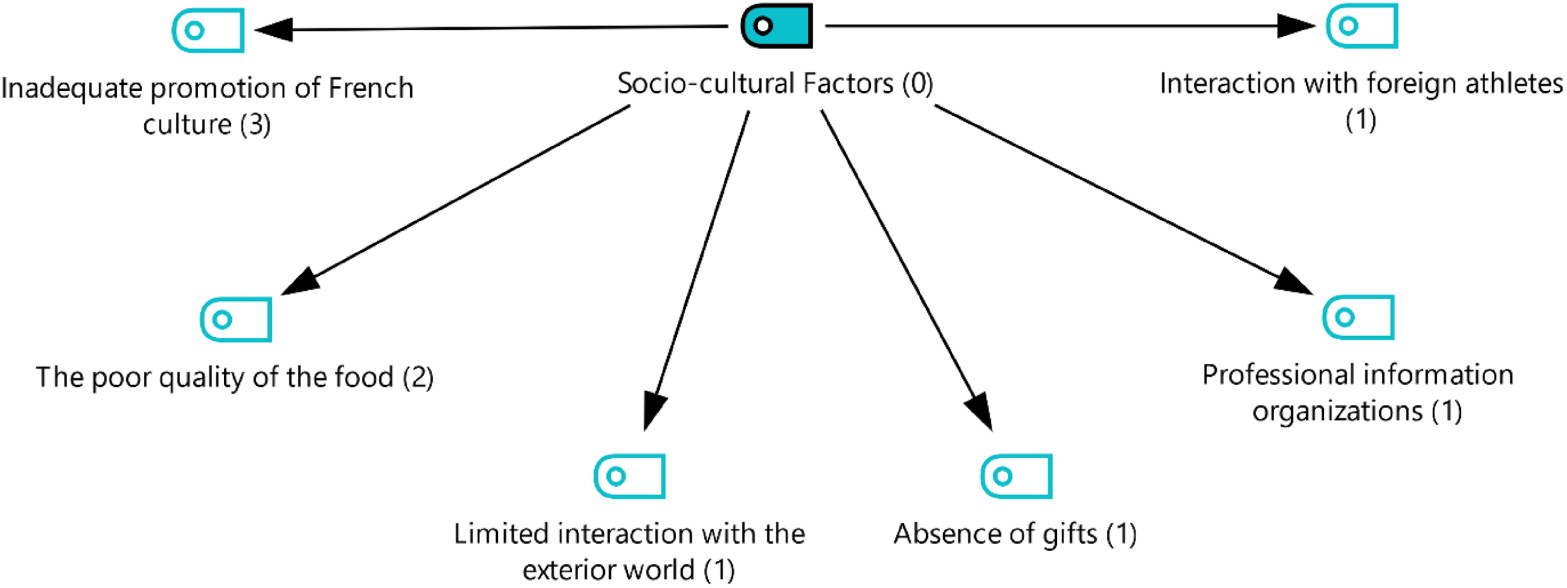
Theme 4—socio-cultural factors.

Under the 4th theme, socio-cultural factors, codes were created for the lack of introduction to French culture, poor food, limited communication with the outside world, lack of gifts, interaction with foreign athletes, and informational events. Participants’ statements related to this theme are listed below (Figure 6):

*“… There was no section dedicated to French cuisine or famous French dishes. Or I would have liked to see something like a display of famous perfumes or a scent stand. (FA3).”*

*“Events usually feature unique, locally specific gifts. Athletes are welcomed with a variety of gifts. We did not see any of these at this mega event (FA3).”*

*“There were some really great arrangements, like having our dietitians there. People we hadn't worked with much before prepared lots of drinks and food for us before the game. Foods that they thought would be beneficial for us. They were a huge support to us in this regard. (FA1).”*

*“I think there was a lot of great interaction there, a very nice atmosphere. Our communication with the foreign athletes was very good (FA1).”*

**Figure 7:**
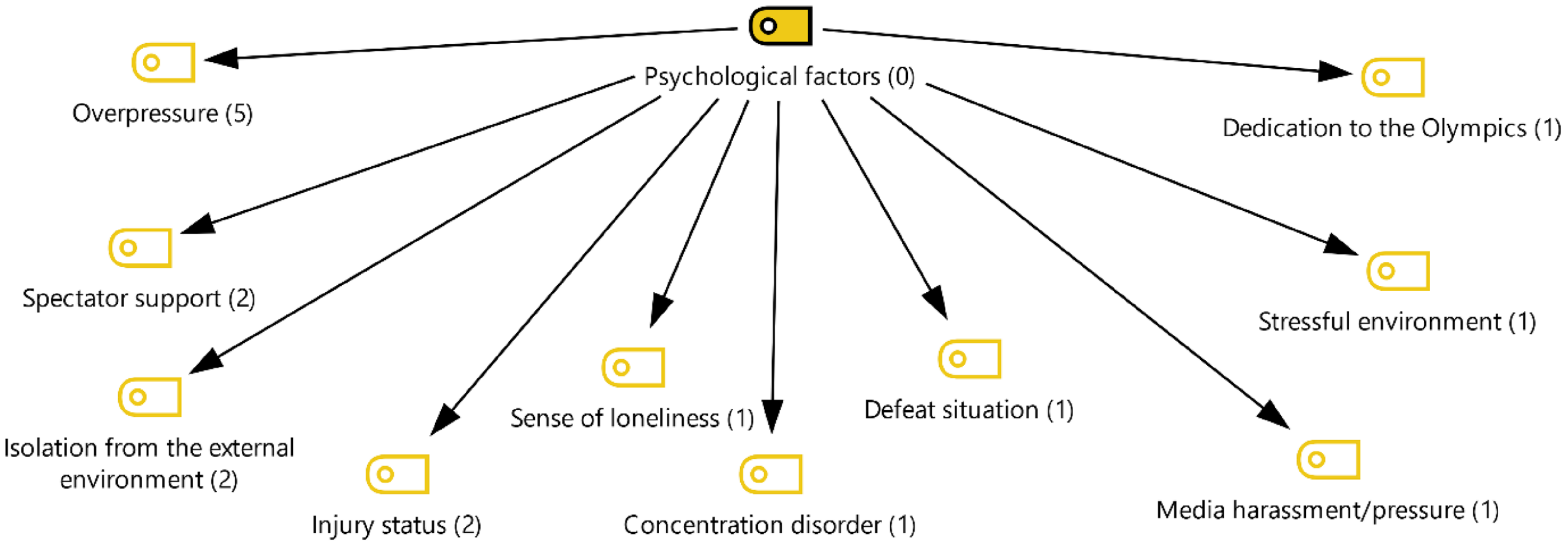
Theme 5—psychological factors.

Figure 7 was created from codes related to psychological factors. Participants evaluated this theme under the headings of excessive pressure, spectator support, isolation from the outside environment, media harassment/pressure, and feelings of loneliness. Some of the statements that formed the codes for this theme are listed below:

*“It's an incredible thing, the spectators recognize all the athletes. They have very successful athletes in every weight class, and there is incredible support. I was very impressed and surprised by this (FA6).”*

*“The Olympic spirit was very much present among the spectators. I saw neutral people supporting the athletes, especially the good ones (FA5).”*

*“My coach wasn't there with me. Going to the Olympics on my own, settling in the village there, it was a constant feeling of loneliness. I didn't feel strong. That's why it affected me so much (FA3).”*

*“I think the biggest problem with my performance was that I couldn't manage the pressure. I think mine was psychological. I was a bit young; I hadn't competed in the Olympics before. Since I was 12, like every athlete, I had the dream of competing in the Olympics… (FA6).”*

*“There was a lot of pressure on me, and I don't think I could handle it. This had a negative impact on my performance (FA2).”*

*“The media could easily reach the places where we were staying. We could see them. That could wear us down psychologically (FA2).”*

*“I'm trying to remember, honestly, the only thing I was thinking about there was the game. I wanted to do it in the best way possible. External factors didn't affect me much (FA4).”*

**Figure 8:**
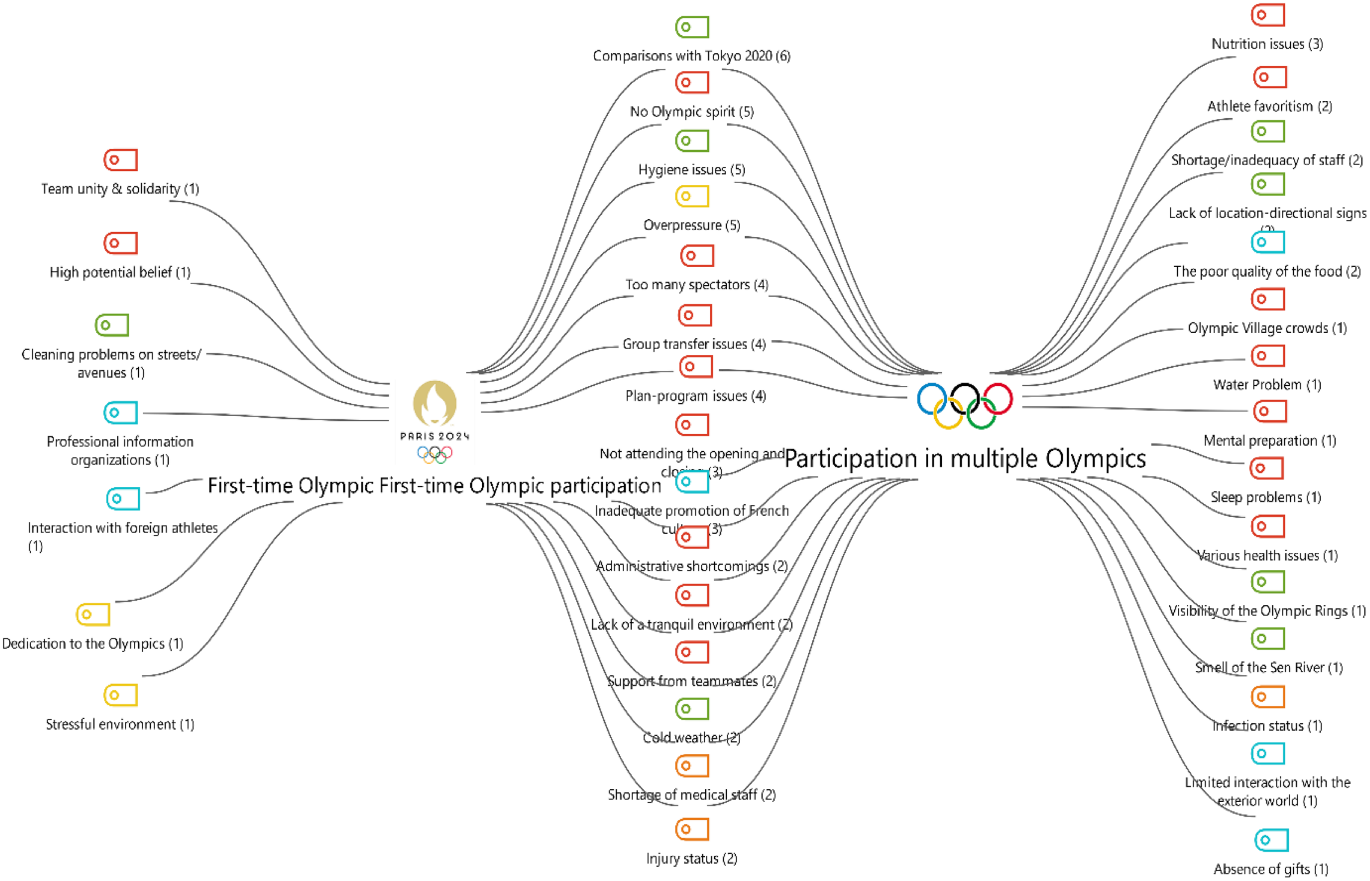
Two-case models.

Two case models created from the perspectives of female athletes participating in the Paris 2024 Olympic Games compare the experiences and challenges faced by athletes participating in the Olympics for the first time with those of experienced athletes who have participated multiple times (Figure 8). The positive aspects that stand out for first-time athletes include team unity and solidarity, high levels of motivation, dedication to the Olympics, and good communication with foreign athletes, while the main challenges encountered include a stressful environment and environmental cleanliness issues. On the other hand, experienced athletes’ comparisons with previous events, particularly Tokyo 2020, show that their expectations are higher. This group has been more critical of the event, highlighting multidimensional issues such as overcrowding in the Olympic Village, infection, hygiene, nutrition problems, health, and adaptation to climate conditions. Looking at the common codes, it is seen that there are negative aspects such as managerial deficiencies, planning and scheduling problems, cold weather, the presence of too many spectators, pressure in the environment, and injuries.

**Figure 9:**
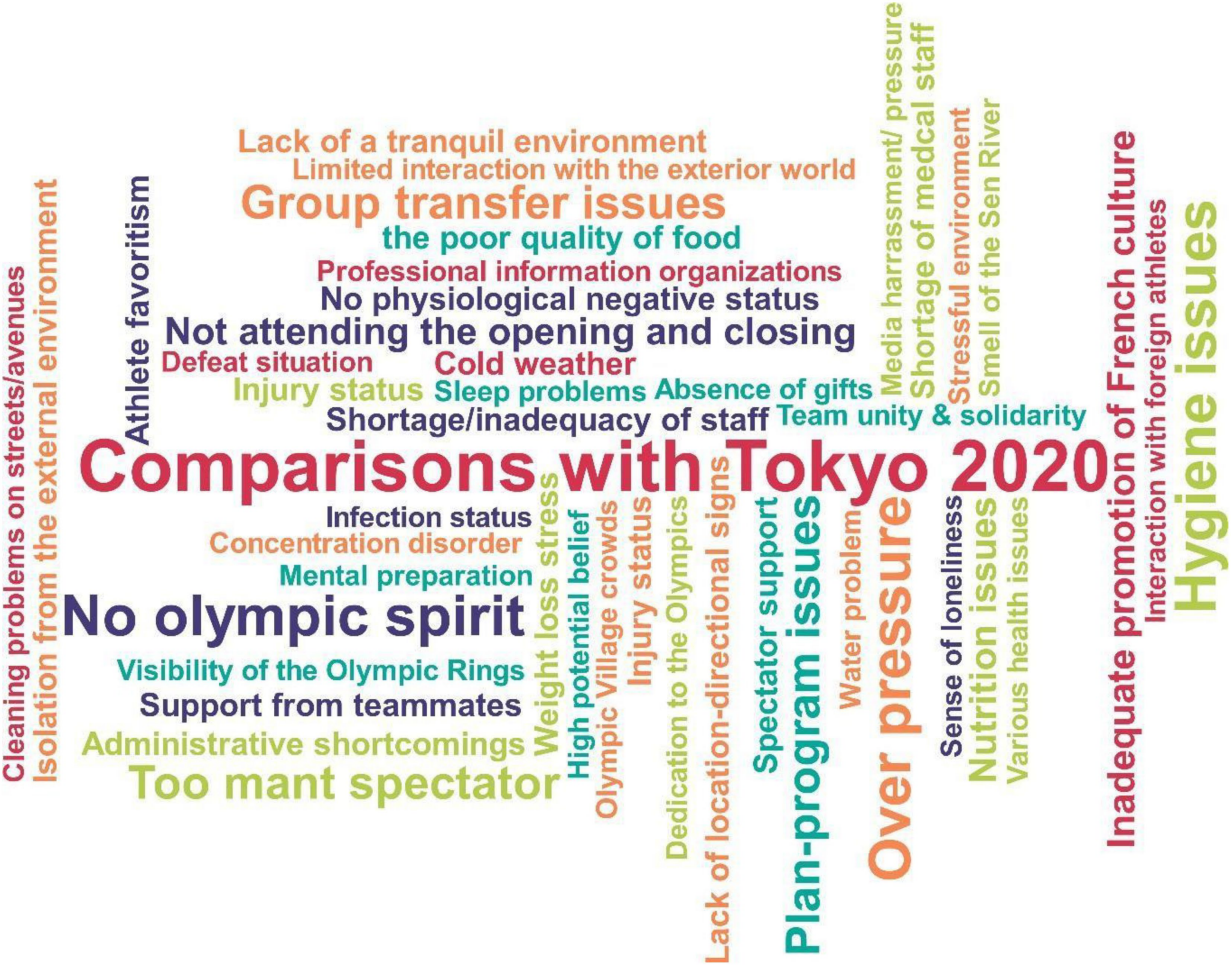
Code cloud.

In the code cloud in Figure 9, when the assessments of Turkish female athletes participating in the Paris 2024 Olympic Games are examined, comparisons with Tokyo 2020 come to the fore. The issues most frequently mentioned by participants include excessive pressure, hygiene problems, lack of Olympic spirit, and the presence of too many spectators. In addition, the fragmented departure of the delegation, planning and scheduling problems, managerial shortcomings, and the inadequacy of officials stand out as significant organizational problems. In terms of health and nutrition, nutrition problems, poor food, insufficient medical staff, weight loss stress, and injuries were frequently mentioned. Furthermore, criticism specific to the host country, such as the insufficient promotion of French culture, the smell of the Seine River, and street cleanliness, was noteworthy. On the other hand, elements such as spectator support and the support of teammates were considered positive experiences by the participants.

## Discussion

This qualitative case study provides insight into the factors affecting the performance of Turkish female athletes participating in the Paris 2024 Olympic Games. The findings were evaluated under five themes: factors most affecting performance, environmental factors, physiological factors, socio-cultural factors, and psychological factors. These themes are consistent with previous studies (Chalkley and Essex, 2010; Tekin and Tekin, 2014) that reveal the Olympic Games are deeply influenced by cultural, political, and environmental elements beyond being merely a sporting platform. Moreover, the themes reveal that the performance of female athletes in their Olympic experiences has a multidimensional and complex structure. Below, each theme is detailed in relation to the literature:

### Factors that most affect performance

Participants indicated that under the first theme, “Factors Most Affecting Performance,” they were affected by the lack of Olympic spirit, spectator density, the fragmented movement of delegations, nutritional issues, and managerial shortcomings. These findings show that in large- scale international sports organizations, not only athletes’ physical abilities but also organizational (Kubiak, 2012) and environmental conditions are decisive for performance (Emery, 1984; Köse, 2025). In this context, the lack of Olympic spirit at Paris 2024 (Hiller, 2002), organizational failures, and the presence of too many spectators were seen to negatively affect athletes’ performance perception. While spectators are a source of motivation for some athletes, they become a source of pressure for others. This situation highlights the importance of many variables in the sustainability of athlete performance.

### Environmental factors

Findings from the environmental factors theme revealed that these elements can directly affect athletes’ experiences and performance. Participants specifically mentioned that factors such as hygiene issues, inadequate signage, and a lack of staff negatively impacted the quality of the organization. Problems related to hygiene and dining areas, in particular, reflect the negative experiences of athletes. The literature indicates that hygiene is important not only in terms of health but also in terms of psychological safety perception (Gursoy and Kendall, 2006). Being in a clean and orderly environment reduces athletes’ stress levels and makes it easier for them to focus on their performance. In addition, lack of guidance in an Olympic village experienced for the first time caused athletes to experience uncertainty, which increased pre-performance anxiety. Thus, Hoye et al. (2015) emphasize that spatial arrangements and guidance tools in sports organizations play a critical role in the participant experience. Another important finding is the inadequacy of staff and deficiencies in support processes. According to athletes’ accounts, the failure to adequately implement the fast and effective staff support provided at Tokyo 2020 created a negative experience at Paris 2024. Chalip (2004) also states that support staff at large- scale sporting events are not only responsible for logistical convenience, but also play a decisive role in terms of hospitality and the perception of safety. Another environmental factor coded as cold weather was found to reduce athletes’ muscle power and force production, increase fatigue rates (Wilmore et al., 2004), and affect athletic performance due to reasons such as the onset of thermogenesis (Lindberg et al., 2012). These findings also align with the environmental factors theme codes.

### Physiological factors

The research findings reveal that the third theme, which includes important physiological factors affecting the performance of female athletes, highlights elements such as inadequate medical support, injury situations, weight loss stress, infection risk, and avoiding physiological disadvantages. The experiences shared by participants show that physiological preparation in sports practice is not limited to training alone, but that medical support, weight management, and injury processes also play a critical role.

First, findings related to healthcare team support highlight the importance of athlete-healthcare team interaction in large organizations. The literature indicates that athletes’ regular work with personal healthcare professionals increases their sense of security and enhances the effectiveness of treatment processes (Andersen, 2011; Waddington and Smith, 2014). However, as seen in the current study, insufficient healthcare team numbers and athletes’ inability to work with familiar specialists can be a factor that increases anxiety levels.

The findings also indicate that weight loss and maintenance pose a significant physiological problem for female athletes. The literature emphasizes that long-term weight control in weight-class sports such as combat sports, wrestling, and weightlifting challenges physiological balance (Franchini et al., 2012; Fogelholm, 1994). The requirement for athletes to maintain a low weight for a certain period of time can lead to both performance loss and health risks. This situation manifests itself, as stated by FA2 in the study, as increased stress in the final period and difficulty in weight loss.

Injuries, on the other hand, cause loss of motivation, lack of physical preparation, and uncertainty before competitions in athletes. Indeed, the experience of FA6 in the study shows that injury before the Olympics creates a double burden, both physiologically and psychologically. Research indicates that injury is not only a physical crisis but also a psychosocial one (Podlog and Eklund, 2007). Therefore, it is evident that athletes need both physiological and psychological support during the injury and subsequent recovery processes.

On the other hand, the fact that some athletes in the study reported experiencing no physiological adverse effects (FA1) highlights the importance of individual differences.

### Socio-cultural factors

The findings of this theme show that the experiences of female athletes demonstrate that mega sporting events such as the Olympics are not only sporting events but also areas of socio- cultural sharing. Participants stated that French culture was not sufficiently promoted and that there was a lack of local food and gifts; on the other hand, they stated that interaction with foreign athletes and informational events were positive experiences.

Firstly, athletes’ expectations regarding the host country’s cultural promotion are directly related to the “cultural heritage and international interaction” dimension of the Olympics. Indeed, the “cultural festival” function of the Olympics is emphasized by the IOC (International Olympic Committee), highlighting the role of sport in enhancing intercultural interaction (Hiller, 2002; Heather et al., 2021). However, the experiences of the participants in the study indicate that this cultural dimension is not sufficiently visible in the organization. The lack of cultural elements may have caused athletes to experience the event solely from a sporting perspective. The participants’ negative experiences regarding meals are also noteworthy. Athletes’ nutrition is important not only for performance but also for cultural adaptation. The literature indicates that the mismatch between the host country’s nutritional culture and athletes’ expectations can cause stress and dissatisfaction (Pelly et al., 2023).

In addition, participants’ attention to the absence of gifts highlights the symbolic aspect of cultural sharing in large-scale events. Local gifts presented to athletes are part of the practices of hospitality and cultural transmission in such events (Minnaert, 2012). The absence of such gifts may have been perceived by athletes as a cultural void.

Another code, participants’ positive references to events aimed at interaction and information sharing with foreign athletes, demonstrates the socio-cultural interaction function of the Olympics. Research emphasizes that communication between athletes from different countries offers important opportunities for cultural learning and the development of social networks (Girginov and Hills, 2023). This shows that some aspects of the organization provide an environment for intercultural interaction.

### Psychological factors

The findings of this theme reveal that the performance of female athletes at the Olympic level is significantly affected by psychological factors. Participants particularly described their experiences under intense pressure, spectator support, feelings of loneliness, media pressure, and isolation from the outside world.

The element of pressure, frequently emphasized by participants, is one of the fundamental psychological stressors affecting athletes’ Olympic performance. The literature shows that athletes feel high levels of pressure in major tournaments such as the Olympics due to historical expectations, national responsibility, and personal goals (Gould et al., 2002; Pensgaard and Duda, 2003; Lu and Zhong, 2023). The statements of FA2 and FA6 in the study also confirm this situation, showing that failure to manage pressure directly leads to loss of performance.

Spectator support was described by participants as both a positive and surprising experience. The statements of FA5 and FA6 indicate that the support of neutral spectators is a motivating factor. Indeed, the literature indicates that spectator support increases self-confidence in athletes and creates a psychological effect similar to the “home advantage” phenomenon (Nevill and Holder, 1999). However, the same support can also cause bewilderment and distraction in inexperienced athletes.

Another factor that stands out in the findings is the feeling of loneliness and isolation. Being separated from FA3’s coach weakened the psychological support mechanism and increased feelings of loneliness. Studies show that athletes’ lack of social support negatively affects their ability to cope with stress and that loneliness has a direct impact on performance (Rees and Hardy, 2000).

In addition, media pressure has been identified as a source of psychological stress for participants. FA2’s statement reveals that media members, who have easy access to athletes’ areas, create psychological distress by violating athletes’ privacy. In the literature, media pressure is considered a factor that increases stress and anxiety levels, particularly among elite athletes (Kristiansen et al., 2011). This situation demonstrates that the media at the Olympics not only makes performance visible but also creates extra pressure on athletes.

### Differences based on experience level

The findings of two case models (Figure 8) that show differences based on the level of experience at Paris 2024 reveal significant differences in the perceptions of first-time participants and experienced athletes. For first-time participants, motivation, team cohesion, and communication with foreign athletes were prominent, while stressful environments and environmental hygiene issues were the main challenges. This situation shows that inexperienced athletes are more sensitive to environmental pressures despite their high motivation (Gould and Maynard, 2009).

Experienced athletes, on the other hand, compared the event to previous ones and stated that their expectations were higher, especially compared to Tokyo 2020, drawing attention to multidimensional issues such as crowds, nutrition, hygiene, health services, and climate adaptation. The literature emphasizes that experienced athletes are more critical of organizational shortcomings due to their past experiences (Wylleman, 2013).

Common challenges for both groups include managerial shortcomings, program disruptions, cold weather, spectator pressure, and injuries. The fact that experienced and first-time athletes face different problems reveals that the Olympic experience directly affects athletes’ perceptions and expectations.

### Research code cloud

The views of Turkish female athletes participating in the Paris 2024 Olympic Games reveal both positive and negative aspects of the experience. Findings indicate that comparisons with Tokyo 2020 are frequently mentioned and that expectations are shaped by this reference point (Kristiansen et al., 2011). Psychologically, the most prominent issues were pressure, spectator intensity, and stress; these factors are also known in the literature to be among the most important psychological stressors affecting performance (Pensgaard and Duda, 2003; Lee et al., 2025). Organizationally, planning and management deficiencies, logistical problems, and staff inadequacies came to the fore. This situation demonstrates that managerial coordination in mega sporting events has an impact on athletes’ experiences. However, spectator support and teammate support have been a strong source of motivation for athletes, and the protective role of social support in coping with stress has once again been revealed (Rees and Hardy, 2000).

## Recommendations

Based on the findings of this study, several recommendations can be made to improve the experiences of Turkish female athletes participating in the Paris 2024 Olympics: Organizational planning and logistical processes must be strengthened, the number of staff increased, and support mechanisms created to respond quickly to athletes’ needs. Psychological and physiological support services should be strengthened; professional teams can be provided to help athletes cope with factors such as pressure, stress, injury, and weight control. Improving environmental conditions and optimizing factors such as hygiene, guidance, and climate will positively affect performance and experience. Furthermore, increasing cultural events and opportunities for international interaction will support athletes’ motivation and satisfaction. Future research could deepen these findings with a larger sample (athletes from different countries and different disciplines). This study could serve as an important guide for sports managers and decision-makers, demonstrating how vital athletes’ multifaceted needs are in the planning and execution of major sporting events.

## Limitations

This qualitative study provides valuable insights into the experiences of Turkish female athletes participating in the Paris 2024 Olympic Games, but it has some limitations. First, as the study is limited to Turkish female athletes, the findings cannot be generalized to data obtained from other countries or male athletes. Participant views are based on subjective statements and may be affected by recall bias and social desirability effects. Furthermore, the findings are specific to the Paris 2024 context and may not be valid for other Olympic Games or sporting events. The researcher’s interpretations and analytical approach during the qualitative analysis process may introduce subjectivity into the findings. Participants’ comparisons with Tokyo 2020 are based on personal perceptions and are not supported by official reports or other sources. Finally, psychological pressure, stress, and physiological conditions were assessed solely through participant statements, without the use of objective measurements. These limitations indicate that caution should be exercised when interpreting the study’s findings.

## Conclusion

This qualitative case study has revealed the multidimensional factors affecting the performance of Turkish female athletes participating in the Paris 2024 Olympic Games. The most significant contribution of this study is that it offers a unique and insider perspective on the Olympic experiences of Turkish female athletes. The findings show that the athletes’ experiences are not limited to sporting performance alone, but are also influenced by environmental, physiological, socio-cultural, and psychological factors.

The study shows that the Olympic experience directly shapes athletes’ perceptions and expectations, and that organizational and environmental shortcomings play a decisive role in performance and psychological state. On the other hand, social resources such as the support of spectators and teammates helped athletes cope with these challenges. The findings also emphasize that the Olympics are an arena for socio-cultural interaction and cultural experience.

The examination of factors affecting the performance of female athletes can yield important results from both a sports science and a gender perspective. At this point, multifaceted variables such as physiological, psychological, environmental, and socio-cultural factors come to the fore. Therefore, discussing the findings is critically important for understanding the success of female athletes in major organizations and developing strategies for the future.

In conclusion, the factors affecting the performance of female athletes are multidimensional and interact with each other. These findings emphasize the importance of comprehensively considering the needs of female athletes in the planning and implementation of international sports organizations. In particular, strengthening health support, nutrition services, cultural adaptation, and social support mechanisms can be recommended as measures to enhance athletes’ performance and experience satisfaction.

## Data Availability

The raw data supporting the conclusions of this article will be made available by the authors, without undue reservation.

## Author’s note

This research was presented as an oral presentation at the 11th Science, Culture, and Sports Congress held in Sharm El Sheikh, Egypt, from April 1–6, 2025.
